# Identification of a multifunctional docking site on the catalytic unit of phosphodiesterase-4 (PDE4) that is utilised by multiple interaction partners

**DOI:** 10.1042/BCJ20160849

**Published:** 2017-02-03

**Authors:** Kirsty F. Houslay, Frank Christian, Ruth MacLeod, David R. Adams, Miles D. Houslay, George S. Baillie

**Affiliations:** 1 Institute of Cardiovascular and Medical Science, College of Medical, Veterinary and Life Sciences, University of Glasgow, Glasgow G12 8QQ, U.K.; 2 Institute of Chemical Sciences, Heriott-Watt University, Edinburgh, U.K.; 3 Mironid Ltd, BioCity Scotland, Bo'Ness Road, Newhouse, North Lanarkshire ML1 5UH, U.K.; 4 Institute of Pharmaceutical Sciences, King's College London, London SE1 9NH, U.K.

**Keywords:** cAMP, cyclic nucleotide phosphodiesterases, protein–protein interactions

## Abstract

Cyclic AMP (cAMP)-specific phosphodiesterase-4 (PDE4) enzymes underpin compartmentalised cAMP signalling by localising to distinct signalling complexes. PDE4 long isoforms can be phosphorylated by mitogen-activated protein kinase-activated protein kinase 2 (MK2), which attenuates activation of such enzymes through their phosphorylation by protein kinase A. Here we show that MK2 interacts directly with PDE4 long isoforms and define the sites of interaction. One is a unique site that locates within the regulatory upstream conserved region 1 (UCR1) domain and contains a core Phe141, Leu142 and Tyr143 (FLY) cluster (PDE4A5 numbering). Located with the second site is a critical core Phe693, Glu694, Phe695 (FQF) motif that is also employed in the sequestering of PDE4 long forms by an array of other signalling proteins, including the signalling scaffold β-arrestin, the tyrosyl kinase Lyn, the SUMOylation E2 ligase UBC9, the dynein regulator Lis1 (PAFAH1B1) and the protein kinase Erk. We propose that the FQF motif lies at the heart of a multifunctional docking (MFD) site located within the PDE4 catalytic unit. It is clear from our data that, as well as aiding fidelity of interaction, the MFD site confers exclusivity of binding between PDE4 and a single specific partner protein from the cohort of signalling proteins whose interaction with PDE4 involves the FQF motif.

## Introduction

The ubiquitous small molecule, cyclic AMP (cAMP), was the first intracellular second messenger to be described and is known to play a pivotal role in regulating many key cellular processes [1–5]. Such cAMP signalling processes are now well understood to be compartmentalised in cells with intracellular gradients of cAMP formed by spatially constrained degradation that is mediated by sequestered populations of cAMP phosphodiesterases (PDEs). Thus, the tethering of particular PDEs to specific signalling complexes, located at distinct intracellular sites, confers a spatial element to cAMP signal processing [1,6,7]. Such intracellular gradients of cAMP are then interpreted by spatially discrete subpopulations of the cAMP effectors, protein kinase A (PKA) and Epac, which differentially regulate different cellular processes [1,5,8,9].

Members of the phosphodiesterase-4 (PDE4) cAMP-specific PDE family play pivotal roles in forming and shaping cAMP gradients within cells. These enzymes are encoded by four genes (*PDE4A*/*PDE4B*/*PDE4C*/*PDE4D*), which generate over 20 distinct isoforms through alternate mRNA splicing and the use of distinct promoters [1,2,10–15]. The fidelity of PDE4 targeting to particular signalling complexes invariably involves specific binding sites that are located within the N-terminal region unique to each PDE4 isoform. It is such precise intracellular targeting that confers specific, non-redundant roles on particular PDE4 isoforms. Such characteristics have been uncovered through the use of dominant negative, siRNA and peptide displacement approaches [16–20]. This has allowed the appreciation of a large and growing collection of partner proteins that are capable of binding PDE4 isoforms: this collection is known as the PDE4 interactome [1].

PDE4 isoforms provide pivotal nodes for cross-talk between the cAMP and other signalling pathways via the ability of particular PDE4 isoforms to be phosphorylated by a range of important protein kinases. Thus, long PDE4 isoforms can be phosphorylated and activated by cAMP-dependent PKA, which provides the key mechanism responsible for cellular desensitisation to cAMP signalling [21–25]. Furthermore, they can be phosphorylated by Erk, MK2, AMPK and an as yet unidentified kinase activated by reactive oxygen/stress pathways [26–30].

The p38 MAPK signalling cascade is a key signal transduction pathway involved in the control of cellular immune, inflammatory and stress responses [31]. Activated p38 MAPK exerts important effects on cell functioning by phosphorylating, and hence activating, the downstream kinase, MK2 [32]. We have previously demonstrated [29] that PDE4 long isoforms can be phosphorylated by MK2. Intriguingly, such phosphorylation appears to have little or no discernible effect on PDE4 activity *per se* [29]. Rather, its effect appears to be targeted to attenuate the stimulatory effect that PKA exerts when it phosphorylates long PDE4 isoforms [29]. In that way, MK2 phosphorylation of PDE4 long forms attenuates the cellular desensitisation to cAMP that stimulatory PKA phosphorylation of PDE4 long forms provides.

PDE4 enzymes from the PDE4B/C/D subfamilies can also be phosphorylated by Erk. This leads to the activation of short forms and, contrastingly, the inhibition of long forms [33]. Interestingly, such modifications require physical association that involves distinct docking and specificity sites that straddle the Erk phosphorylation site located within the PDE4 catalytic unit [33].

Here, we show that MK2, like Erk, interacts directly with PDE4 through distinct docking sites. One such docking site is located, along with the phosphorylation site, in the regulatory upstream conserved region 1 (UCR1) that is unique to PDE4 long isoforms. We also uncover an additional docking site, which is located within the PDE4 catalytic unit, which is employed by an array of proteins capable of interacting with PDE4. We propose that this forms a multifunctional docking site (MFD) that, as well as aiding fidelity of interaction, confers exclusivity of binding between PDE4 and specific partner proteins.

## Materials and methods

### SPOT synthesis of peptides and peptide array probing

Peptide libraries were generated by automatic SPOT synthesis on Whatman 50 cellulose membranes using Fmoc (9-fluorenylmethyloxycarbonyl) chemistry with the Autospot-Robot ASS 222 (Intavis Bioanalytical Instruments, Koeln, Germany). The interaction of peptide spots with GST and GST-fused purified proteins by overlaying the cellulose membranes with 10 mg/ml recombinant protein was determined as described previously in detail [16,34,35]. Bound recombinant proteins were detected with specific primary antisera and complementary HRP-coupled secondary antibody as for immunoblotting.

### Cell culture and preparation of lysates

COS-1 and HEK (human embryonic kidney)293 cells were propagated as previously described [17,36]. Cell lysate for western blotting or immunoprecipitation was prepared using the protocol outlined in ref. [29].

### Cell transfection

PolyFect® (QIAGEN®) was used for transfection of COS-1 and HEK293 cell lines as previously described [29].

### Co-immunoprecipitation

Immunoprecipitations were carried out as previously described in refs [29,34] using a specific antibody against MK2 (Cell Signaling Technology, Cat. no. 3042).

### SDS–PAGE

Protein samples were separated on NuPAGE 4–12% Bis–Tris polyacrylamide gels (Invitrogen, U.K.) as previously described [29].

### Western blotting

The proteins separated by SDS–PAGE were transferred to a nitrocellulose membrane for western blotting using the procedure outlined previously [29]. The commercially obtained primary antibodies used in this study were against MK2 (Cell Signaling Technology, Cat. no. 3042), P38 (Cell Signaling Technology, Cat. no. 9212), phospho-P38 (Cell Signaling Technology, Cat. no. 9215), p44/42 ERK MAPK (Cell Signaling Technology, Cat. no. 4696), β-Arrestin1 (Cell Signaling Technology, Cat. no. 4674) and GST (Cell Signaling Technology, Cat. no. 2624). The antibodies to PDE4A5, PDE4B, PDE4C, PDE4D and phospho-PDE4A5 were raised by the Baillie Laboratory and have been described previously [18,22,29]. Arrestin and ERK were detected concomitantly during competition overlay experiments on peptide arrays using the Licor Odyssey Scanner as previously described [16].

## Results and discussion

### MK2 co-immunoprecipitates with PDE4 long isoforms

We have previously shown [29] that activated MK2 phosphorylates the long PDE4A5 isoform at a single site, namely Ser147, which is located within the regulatory UCR1 domain that characterises PDE4 long isoforms from all four subfamilies [1,10,13] where this residue and the surrounding MK2 consensus phosphorylation motif are conserved.

**Figure 1.**
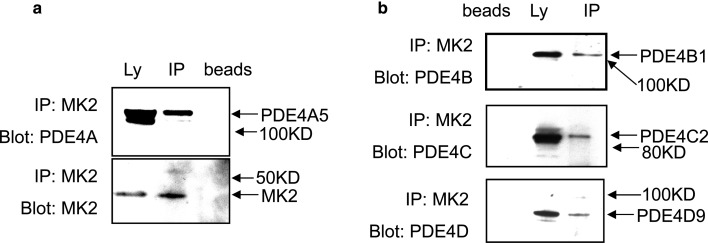
Co-immunoprecipitation of PDE4A5 with MK2 from mammalian cells. (**a**) COS-1 cells were transiently transfected to express the long PDE4A5 isoform. Endogenous MK2 was then immunoprecipitated using specific antisera (Cell Signaling Technology, Cat. no. 3042). The lysates (Ly) and immunoprecipitates (IP) were each probed with either MK2-specific antisera (lower panel) or rodent PDE4A-specific antisera (upper panel). (**b**) Cos-1 cells were transiently transfected to express the long isoforms of PDE4 as indicated. Endogenous MK2 was immunoprecipitated using the MK2-specific antisera. The immunoprecipitates and lysates were each probed with antisera specific for PDE4B, PDE4C or PDE4D, as indicated. These identified the co-immunoprecipitation of the endogenous long PDE4 Isoforms, PDE4B1, PDE4C2 and PDE4D9. Also shown are the probing of the ‘bead-only’ lanes where no MK2-specific antisera were employed as a control. These data show typical experiments of ones performed three times.

For the MAP kinase, Erk to phosphorylate authentic cellular substrates, such as PDE4, it binds directly to them via docking and specificity sequences that lie on each side of the phosphorylation site [33]. With this in mind, we set out to investigate whether MK2 might bind to PDE4A5 in a similar fashion. Thus, we first transfected Cos1 cells with the long PDE4A5 isoform in order to determine whether this ectopically expressed PDE isoform could bind to endogenous MK2. Lysates from such transfected cells were then subjected to immunoprecipitation, using a pan rodent PDE4A isoform, which allowed us to demonstrate that PDE4A5 does indeed co-immunoprecipitate together with MK2 (Figure 1a).

We then subjected lysates of Hek293 cells to immunoprecipitation using MK2-specific antisera and probed these, together with the lysates, with antisera specific for each of the PDE4B, PDE4C and PDE4D subfamilies (Figure 1b). This allowed us to demonstrate that endogenous long isoforms from each of these three PDE4 subfamilies were capable of co-immunoprecipitating with endogenous MK2 (Figure 1b). Note that any putative endogenous PDE4A isoforms that might be expressed in these cells are below levels detectable by our antisera.

### Peptide array analyses to locate MK2-binding sites on PDE4A

We, and others, have successfully used a peptide array approach to delineate various protein–protein interaction surfaces (see, for example, [1,34,35,37]). This provides a novel and powerful technology for gaining insights into the basis of specific protein–protein interactions and driving intelligent mutagenesis approaches to confirm or refute binding site authenticity in the intact protein. We thus utilised this strategy to identify the sites within PDE4A5 that MK2 binds to.

**Figure 2.**
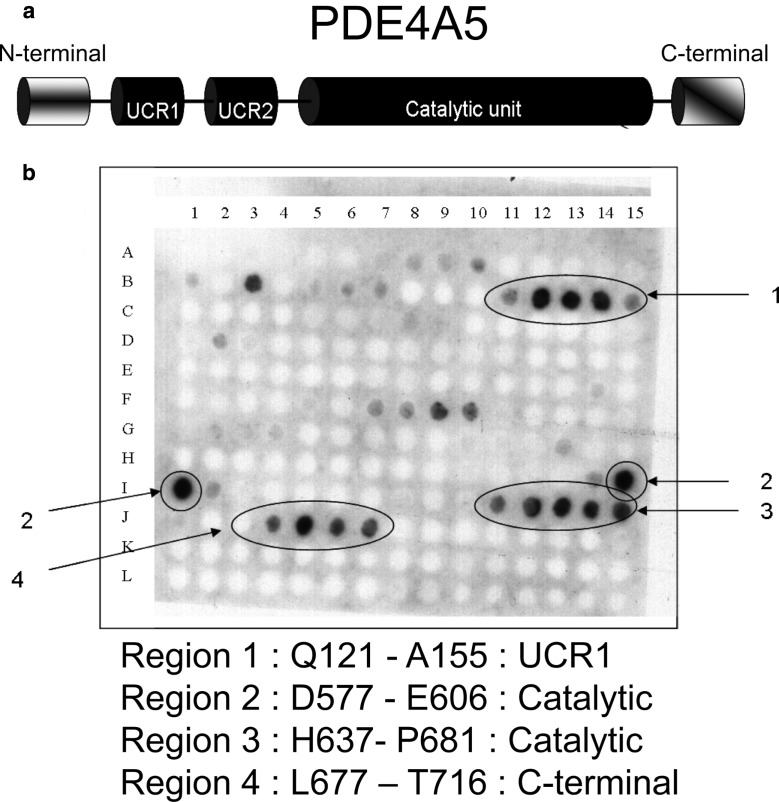
The binding of MK2 to PDE4A5 peptide array. Peptide array technology was used to synthesise a full-length array of the entire 844 amino acids forming PDE4A5 on a Whatman 50 membrane (amino acids identified by numbering from sequence with accession number P54748). A schematic representation of the modular structure of PDE4A5 is shown in (**a**). The PDE4A5 array consisted of consecutive 25 amino acid peptide spots that each overlapped by five amino acids. The membrane was probed with purified recombinant GST-tagged MK2 (**b**). GST-specific antisera were then used to detect the areas of MK2 binding to the membrane. Highlighted are the four main regions of most intense binding and their sequences. All peptide arrays are representative arrays of at least three separate experiments.

A full-length peptide array of PDE4A5 was made using procedures utilised by us previously to evaluate the interaction of other PDE4 isoforms with partner proteins [16,34,35,38]. This consisted of a library of peptide ‘spots’ of 25 amino acids that sequentially span the PDE4A5 sequence and where each peptide overlapped the previous peptide by five amino acids. This array was then overlaid with purified recombinant GST-tagged MK2. The location of putative binding sites was determined by probing with GST-specific antisera and processed in a similar fashion to a western blot as described previously in detail [1,34,35]. Such analyses showed that when spots previously shown to bind GST alone were discounted, then four potential binding sites for MK2 on PDE4A5 were identifiable (Figure 2). These sites are associated with amino acids 121–155 located within UCR1, amino acids 577–606 and 637–681, both located within the catalytic region, and amino acids 677–716 located within the C-terminal region (Figure 2).

**Figure 3.**
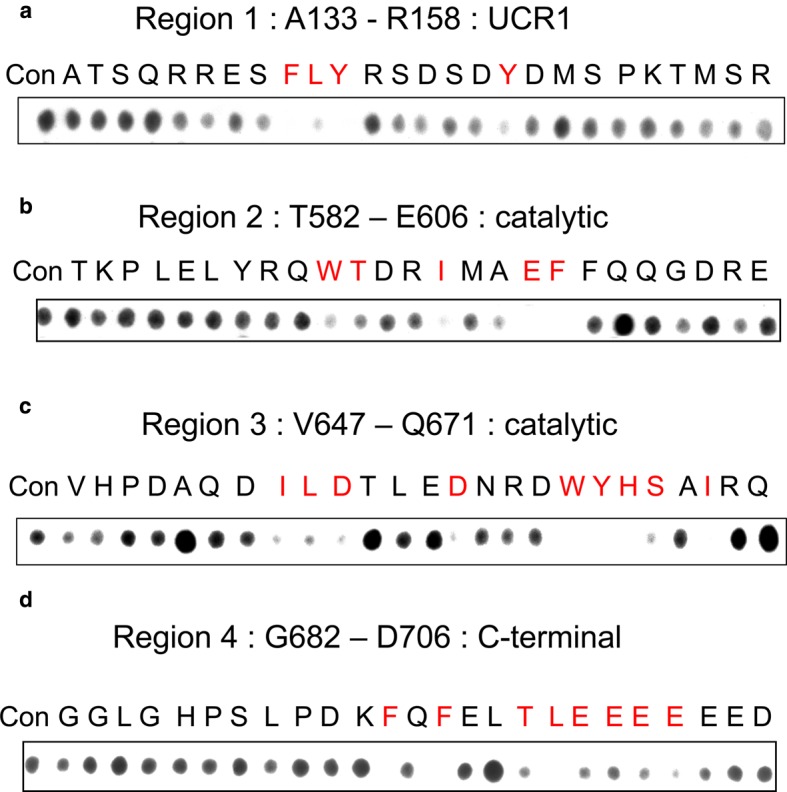
Alanine scanning analyses of MK2-binding site(s) on PDE4A5. Peptide array technology was used to synthesise alanine scanning arrays of PDE4A5 on a Whatman 50 membrane. This was done using specific sequences indicated. These are amino acids (**a**) 133–158, (**b**) 582–606, (**c**) 647–671 and (**d**) 682–702. A control spot was created with each sequence and each amino acid in turn was mutated to alanine. The membrane was probed with purified recombinant GST-tagged MK2. GST-specific antisera were then used to detect the areas of MK2 binding to the membrane. Highlighted in red are the main amino acids where substitution with alanine attenuates binding. All peptide arrays are representative arrays of at least three separate experiments.

To gain further insights into particular amino acids that are important in each of these regions and to confirm the fidelity of interaction at such sites, alanine scanning peptide arrays were created for part of each region identified above. In undertaking this, we screened a family of peptides derived from distinct 25-mer parent peptides that positively interacted with MK2. As utilised previously in studies of other protein–protein interactions [1,34,35], the peptide progeny from specific 25-mer parents was generated such that each had a single substitution, to alanine, of successive amino acids in the sequence to form a scanning peptide array. If alanine residues existed in the sequence to be scanned, these were substituted with an aspartate. The alanine scanning arrays were each probed with purified recombinant GST-tagged MK2 (Figure 3).

Examining the potential MK2 interaction site within the regulatory UCR1 of PDE4A5, we see that substitution, to alanine, of any of the amino acids within the cluster of Phe141, Leu142 and Tyr143 essentially ablated interaction (Figure 3a). Binding was also compromised with several other alanine substitutions of amino acids within this region, which was particularly evident for Tyr149 (Figure 3a). In the first catalytic region sequence, amino acids identified as being potentially required for MK2 binding by alanine scanning peptide analysis were Trp591, Thr592, Ile595, Glu598 and Phe599 where alanine substitution compromises MK2 interaction (Figure 3b). In the second catalytic region sequence, amino acids identified as being potentially required for MK2 binding by alanine scanning peptide analysis were Ile654, Leu655, Asp656, Trp664, Tyr665, His666, Ser667 and Ile669 (Figure 3c). Finally, in the C-terminal region, amino acids identified as being potentially required for MK2 binding by alanine scanning peptide analysis were Phe693, Gln694, Phe695, Thr698, Leu699, Glu700, Glu701, Glu702, Glu703 and Glu704 (Figure 3d).

**Figure 4.**
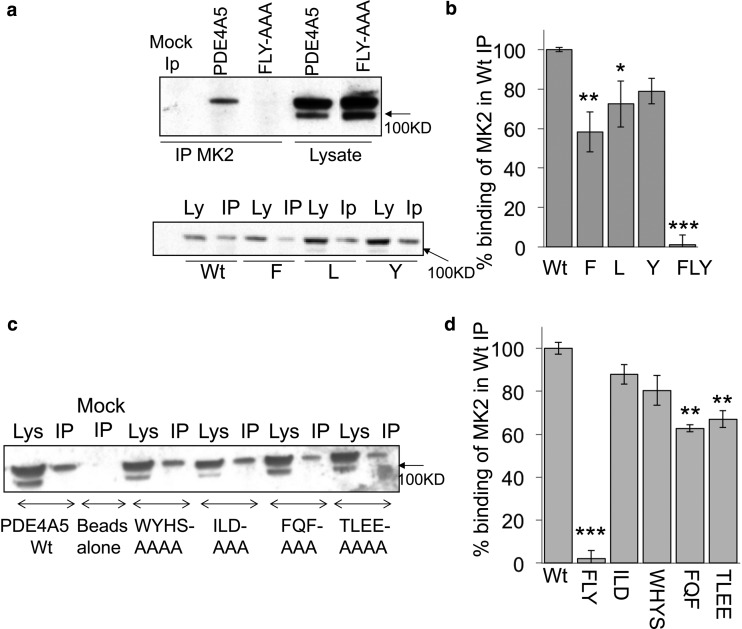
Co-immunoprecipitation of PDE4A5 and mutant species with MK2. PDE4A5-binding sites for MK2 interaction were mutated as indicated and expressed in COS-1 cells. Immunoprecipitations of endogenous MK2 were prepared from cellular lysates and the co-purification of PDE4A5 and PDE4A5 mutants was assessed by western blotting using PDE4A5-specific antibody. The specific PDE4A5 mutants tested were (**a**) the FLY motif, (**b**) individual amino acids within the FLY motif and (**c**) other motifs that had been identified as being important for the MK2 association (Figure 3). Quantification of the importance of the FLY (Phe141, Leu142, Tyr143 cluster), ILD (Ile654, Leu655, Asp656 cluster), WHYS (Trp664, Tyr665, His666, Ser667 cluster), FQF (Phe693, Gln694, Phe695 cluster) and TLEE (Thr698, Leu699, Glu700, Glu701 cluster) motifs are shown in (**d**). Significances of mutations evaluated using Student's *t*-test compared with wt PDE4A5. *P* < 0.05 (*), *P* < 0.01 (**), *P* < 0.01 (***).

To evaluate the potential importance of such regions for PDE4A5–MK2 interaction, we adopted a mutagenesis strategy (Figure 4). In this we focused on, first, the Phe141, Leu142 and Tyr143, which we call the FLY motif, located in the regulatory UCR1 domain conserved in all PDE4 long isoforms and where the MK2 phosphorylation site is located (Figure 4a). Interestingly, individual mutations of any single one of these amino acids to alanine partially compromised binding, while the triple alanine mutation of all three residues fully ablated interaction, uncovering the pivotal importance of the FLY motif in allowing MK2 to bind to PDE4A5 (Figure 4b).

Unlike for UCR1, the structure of the PDE4A catalytic unit is known [39]. Thus, we adopted our previous strategy for mapping binding sites [34], namely to focus on residue clusters identified from alanine scanning peptide arrays that have surface availability (Figure 4b). In the PDE4A catalytic unit, these are the Ile654, Leu655, Asp656 cluster; the Trp664, Tyr665, His666, Ser667 cluster; the Phe693, Gln694, Phe695 cluster and the Thr698, Leu699, Glu700, Glu701 cluster. We generated mutants of PDE4A5 for each of these clusters separately, where all of the residues in each cluster were mutated in their entirety to alanine. Such PDE4A5 mutant constructs were individually expressed at similar levels in COS-1 cells, which do not express detectable levels of endogenous PDE4A isoforms. Lysates were then generated and MK2 immunoprecipitates probed for the presence of PDE4A5 (Figure 4a,b). These experiments showed that, in marked contrast with native PDE4A5, a PDE4A5 construct with a triple alanine mutation of the FLY domain, namely FLY:AAA-PDE4A5, failed to co-immunoprecipitate with MK2 (Figure 4a). Additionally, alanine mutation of each of the catalytic site clusters examined here showed compromised interaction with PDE4A5, with the FQF:AAA-PDE4A5 and TLEE:AAAA-PDE4A5 species showing the most severe reduction in interaction (Figure 4b). The attenuated binding of the mutated PDE4A5 species highlights the importance of these docking domains in maintaining the fidelity of complex formation with MK2. Nevertheless, the possibility has to be entertained that these reductions could be a result of a dramatic change in conformation of the PDE protein elicited by such discrete mutations.

### FLY domain is essential for phosphorylation of PDE4A5 by MK2

We have demonstrated previously [29] that MK2 can phosphorylate PDE4A5 on residue Ser147 that is located within UCR1. Such a modification serves to attenuate the activation of PDE4A5 that is engendered via PKA phosphorylation of residue Ser140 [22], which is also located within UCR1.

**Figure 5.**
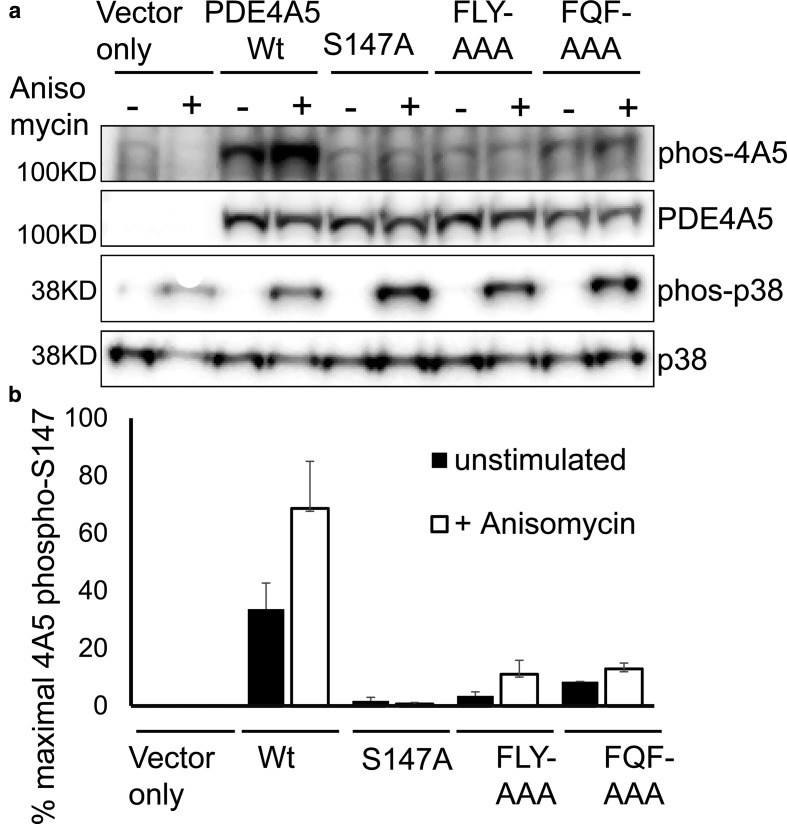
Mutants of PDE4A5 that are compromised in MK2 binding show reduced phosphorylation on serine 147 following anisomycin treatment. (**a**) HEK293 cells were transiently transfected with plasmids encoding the indicated PDE4A5 variants or empty vector. p38 MAPK was activated by a 1 h stimulation using anisomycin. Cell lysates were probed using specific antisera for total and phosphorylated (Ser147) PDE4A5 and for total and phosphorylated p38 MAPK (Thr180, Tyr182). The blot shown is a representative result for *n* = 4. (**b**) Quantification of PDE4A5 (Ser147) phosphorylation of PDE4A5 wt and mutants following anisomycin treatment (*n* = 4).

In the present study, we have shown that MK2 docks with PDE4 and identified putative docking sites for MK2 on PDE4A5. We thus surmised that, as for Erk interaction with PDE4 and other substrate proteins, PDE4A5 mutants showing reduced MK2 binding might also exhibit compromised phosphorylation by MK2 on Ser147. We set out to examine this using the well-established approach of using anisomycin to promote activation of the p38 MAPK pathway and, thus, of MK2 [31]. As shown previously by us [29], anisomycin challenge of COS-1 cells expressing PDE4A5 promoted its phosphorylation at Ser147 by MK2 and this was ablated in PDE4A5 mutants where the target for phosphorylation, namely Ser147, was substituted with alanine (Figure 5; Ser147Ala-PDE4A5 mutant). Intriguingly, both the FLY:AAA-PDE4A5 and FQF:AAA-PDE4A5 mutants showed marked reductions in the anisomycin-driven Ser147 phosphorylation when compared with wild type (wt, Figure 5). These observations demonstrate that the interaction of PDE4A5 with MK2, through its two distinct docking sites, facilitates the efficient phosphorylation of PDE4A5 at Ser147.

### FQF lies at the core of an MFD site

The FLY motif appears to be essential for MK2 binding to PDE4A5. However, as is the case with all known PDE4 partner proteins, fidelity of interaction appears to be driven through the involvement of more than one binding site [1]. Invariably, this allows the partner protein to straddle the catalytic unit, with one site in either the regulatory domains (UCR1/2) or the isoform-specific N-terminus and another in the catalytic unit. Interestingly, the Phe693:Gln694:Phe695 (FQF) motif on the catalytic unit of PDE4A5 that we have identified here as contributing to MK2 binding also is intimately involved in the binding of Erk [33], β-arrestin [34], Lis1 (PAFAH1B1) [38] and AKAP18 [40]. This suggested that FQF is located at the core of an MFD site that confers fidelity on partnerships by preventing multiple partner proteins to interact with any one PDE4 isoform simultaneously. Indeed, we have shown that competition exists between different binding partners of PDE4 for the FQF domain using simultaneous overlay of multiple purified proteins onto PDE4 peptide arrays [16].

**Figure 6.**
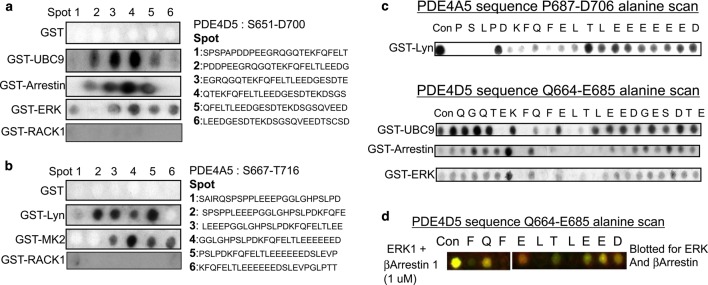
Delineation of an MFD site on long-form PDE4 enzymes. Peptide array technology was used to synthesise peptide arrays of PDE4D and PDE4A5 regions encapsulating the FQF domain. This was done using specific sequences indicated. These are amino acids (**a**) 651–700 of PDE4D5 and (**b**) 667–716 of PDE4A5. The PDE4D5 arrays were overlaid with GST, GST-UBC9, GST-Arrestin and GST-ERK2, whereas the PDE4A5 arrays were overlaid with GST, GST-Lyn and GST-MK2. GST-specific antisera were then used to detect the areas of protein binding to the membrane. (**c**) Peptide array technology was used to synthesise alanine scanning arrays of the PDE4A5 and PDE4D5 regions encapsulating the FQF domain. This was done using specific sequences indicated. These are amino acids on upper panel PDE4A5, 687–706 and lower panel PDE4D5, 664–685. A control spot was created for each sequence and each amino acid in turn was mutated to alanine. All peptide arrays are representative arrays of at least three separate experiments. (**d**) Equal amounts (1 µM) of GST-βArrestin 1 and GST-ERK1 were simultaneously applied to alanine scanning arrays of the PDE4D5 region encapsulating the FQF domain. Arrestin and ERK were detected concomitantly using the Licor Odyssey Scanner as previously described [16].

PDE4A5 was reported some time ago to bind to the SH3 domain of Lyn tyrosyl kinase [41,42] and, additionally, we have shown that PDE4 isoforms can functionally interact with the SUMOylation E2 ligase, UBC9 [43]. We thus set out to investigate whether the FQF motif might be highlighted in PDE4 peptide arrays probed with these two species as well as with other binding partner proteins that we have reported on, namely β-arrestin and ERK [27,33] (Figure 6).

Lyn and MK2 appear to interact specifically with PDE4A rather than isoforms of the other three PDE4 subfamilies [44]. The C-terminal regions of all four subfamilies are distinct, with no regions of homology. Indeed, such a difference has been exploited to make subfamily specific antisera [45]. Thus, we set out to probe peptide arrays representing the C-terminal region of PDE4A5 with purified Lyn, UBC9, Arrestin2 and ERK2 (Figure 6a,b). These data show that all these very different proteins were capable of binding to PDE4A5 peptides containing the FQFELTL sequence; however, RACK1, a protein that is known to bind elsewhere in the catalytic unit, did not (Figure 6a,b).

Alanine scanning peptide arrays were created for each region and probed with the PDE4-binding partners as before (Figure 6c). In all cases, the dual phenylalanines and leucines within the FQFELTL motif were shown to be crucial for partner binding to the PDE4 sequence. This suggests a common mode of interaction employed by all these very different proteins in the way that they bind to this MFD platform. As before [16], we show using competition overlay on alanine scans of the MFD motif that both ERK1 and β-Arrestin1 compete for the wt peptide (arrestin in green, ERK in red and dual binding in yellow; Figure 6d) and that substitution of the dual phenylalanines and leucines is equally effective in ablating the binding of both PDE4-interacting proteins. We envisage that such binding may serve to aid in orientating such proteins to locate additional, partner-specific binding sites on the PDE4 host.

**Figure 7.**
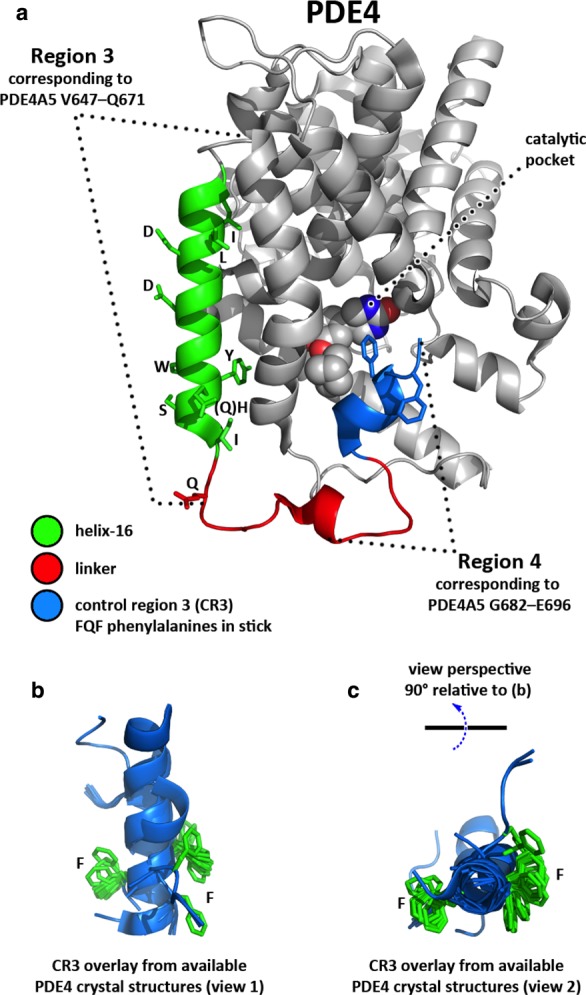
Structure of the PDE4 MFD domain and candidate interaction site on MK2. (**a**) The PDE4 core catalytic domain is shown from PDE4B co-crystal structure 3KKT with the bound inhibitor (spheres) marking the catalytic pocket. The Region 3–MK2 interaction sequence of PDE4A5 is shown mapped onto the structure, with specific interaction residues implicated in the array study shown (stick). The Region 4 peptide is similarly shown, spanning the C-terminal linker (red) and CR3 helix (blue), as far as the currently available crystallographic limit. The FQF phenylalanines are shown (blue stick) with CR3 in a capped position across the catalytic pocket. (**b**) In the majority of PDE4 crystal structures, the C-terminal linker and CR3 are disordered; an overlay of the CR3 peptides from those structures that exhibit some order reveals helical secondary structure but with a degree of conformational variability, especially affecting the presentation of the second Phe in the FQF motif (which lies at or near the limit of ordered structure determined to date). (**c**) An orthogonal view of the CR3 overlay is shown relative to that in (**b**).

Interestingly, the structure of the proposed MFD site can be identified from observations of several PDE4 crystal structures as located within the so-called CR3 region [46–53]. In these PDE4-only structures, the MFD site can be seen as lying across the opening of the catalytic pocket (Figure 7a), typically through insertion of a single phenylalanine from the FQF motif. Although some variation is seen in the mode of interaction with the core catalytic domain in these structures, it has been proposed that this crystallographically observed capture of CR3 by the catalytic pocket might reflect an autoinhibitory capping role that is relevant to PDE4 function [49]. In all structural determinations, to date, the CR3 region containing the FQF motif exhibits helical secondary structure, although some conformational variability is seen across the available crystal structures (Figure 7b,c). Thus, this region appears to be positionally mobile with respect to its point of attachment (helix-16) on the PDE4 core catalytic domain, which would be consistent with an ability to associate with appropriate partner proteins.

The binding of the CR3 region across the PDE4 catalytic pocket typically involves insertion of one of the two Phe residues of the FQF motif into the catalytic pocket. As such, one of the Phe residues would be inaccessible to a partner protein, whereas the other Phe would be solvent-exposed and therefore accessible to a partner protein. In principle, then partner protein binding might potentially occur with this region in such a capped position. However, the results of the peptide array analyses on PDE4A5 with arrestin2, UBC9, ERK, Lyn and MK2 suggest that, for maximal interaction, both Phe residues are likely to be involved in the binding of these partner proteins. The implication of both Phe residues being involved in partner protein binding with PDE4 is that the MFD sequence would be removed from any association with the catalytic pocket when such partner proteins bind to PDE4. This would be fully consistent with the flexible, hinge-like nature of CR3.

## Conclusion

Specifically sequestered PDE4 isoforms play a major role in underpinning the compartmentalisation of cAMP signalling cells [1]. Associated with this, such species also provide nodes of cross-talk between a wide range of signalling pathways that is achieved through the multisite phosphorylation of PDE4 isoforms [1,54]. The p38 MAPK phosphorylation cascade plays a fundamental role in regulating the immune and inflammatory response to infection and tissue injury [31]. MK2, the downstream effector kinase of p38 MAPK, has been shown [29] to play a key role in attenuating cellular desensitisation to cAMP by phosphorylating PDE4 long isoforms such that their ability to be activated by positive feedback PKA phosphorylation [23] is diminished. Here, we show that in order for MK2 to phosphorylate PDE4 long isoforms effectively, this kinase interacts with docking sites located on PDE4. Such a docking and consequential phosphorylation process is akin to that employed by Erk when it phosphorylates PDE4 [33] and other physiological substrates, each of which is defined by the presence of a conserved docking site [55,56].

PDE4 enzymes thus have binding domains that allow interaction with various kinases and domains that target them to scaffold proteins. These allow for the spatially discrete degradation of cAMP so as to confer compartmentalised signalling, which can then be regulated through cross-talk with other signalling processes [1].

The ability to form functionally discrete cAMP signalling complexes (signalosomes) involving a specific anchor protein and a specific PDE4 isoform will thus depend on the fidelity of partnerships. However, various signalling scaffold proteins are capable of interacting with a wide range of all PDE4 isoforms. In such instances, if a particular PDE4 isoform was able to interact with more than one scaffold at a time, then this would probably lead to multimerisation into aggregates with loss of integrity of spatial compartmentalisation of cAMP degradation. Similarly, the ability of PDE4 enzymes to be phosphorylated by various kinases will depend on the availability of free docking sites. Here, through our analyses of the interaction of the functionally important long PDE4A5 isoform, we have identified one means through which multimerisation through interaction with multiple scaffolds is prevented and availability for modification by a specific regulatory kinase is effected, namely through the identification of a cohort of interaction proteins that require docking to a common site, the MFD site.

The sequestration of PDE4 isoforms by signalling scaffolds invariably involves binding at two or more sites on the PDE4 [1]. If partnerships are either isoform-specific or show preference for one particular isoform, then one binding site is located within the isoform-specific N-terminal region. However, various PDE4 isoforms are capable of interacting with a range of signalling proteins with scaffolding attributes. Here, we have identified an MFD site on PDE4A5, which is conserved in all PDE4 isoforms from all four subfamilies, that provides one of the essential sites used by a cohort of signalling proteins to interact effectively with PDE4 enzymes, namely the protein kinases MK2 and ERK, the signalling scaffold proteins β-arrestin and Lis1 (PAFAH1B1), the SUMO E2 ligase UBC9, and the tyrosyl protein kinase and scaffold protein Lyn. The requirement of a scaffold protein/kinase for binding to such an MFD would indicate that PDE4 subpopulations sequestered by one scaffold using this MFD would be unable to interact with other scaffolds using this MFD, hence ensuring spatial fidelity. Furthermore, when a PDE4 isoform interacts with a protein partner via the MFD, the PDE4 enzyme is unlikely to be effectively phosphorylated by a kinase that also requires the MFD.

Thus, interaction of PDE4 isoforms with MFD-binding partner proteins will define not only subpopulations that are spatially distinct but also that show attenuated susceptibility to phosphorylation by kinases such as MK2 and Erk, both of which modify the functional outcome of stimulatory PKA phosphorylation [28,29].

## Abbreviations

cAMP: cyclic AMP
HEK: human embryonic kidney
MFD: multifunctional docking
MK2: mitogen-activated protein kinase-activated protein kinase-2
PDE4: phosphodiesterase-4
PKA: protein kinase A
UCR1: upstream conserved region 1
wt: wild type.
